# Population-Level Analysis of Personalized Food Recommendation Using Reinforcement Learning

**DOI:** 10.3390/foods14213770

**Published:** 2025-11-03

**Authors:** Yone Tellechea, Markel Arrojo, Ander Cejudo, Cristina Martin

**Affiliations:** 1 Vicomtech Foundation, Basque Research and Technology Alliance (BRTA), Mikeletegi 57, 20009 Donostia-San Sebastián, Spain; 2 Faculty of Engineering, University of Deusto, Avda. Universidades 24, 48007 Bilbao, Spain; 3 e-Health Department, Biodonostia Health Research Institute, Paseo Dr Begiristain s/n, 20014 Donostia-San Sebastián, Spain

**Keywords:** food recommender systems, consumer preferences, entire food supply chain, waste reduction

## Abstract

This paper introduces an innovative methodology for optimizing recommendation strategies across different populations within the food industry. While previous approaches to recommending courses have overlooked cultural and age-based preferences, our work demonstrates how understanding these differences can significantly enhance the attractiveness for consumers and create new opportunities for marketing. By simulating diverse populations using a fuzzy logic approach, based on individual characteristics such as age, gender, geographical area, and city size, the study evaluates how recommendation algorithms perform within a generated menu database. Results show that algorithms like State–Action–Reward–State–Action (SARSA), multi-armed bandit (MAB), and Deep-Q Network (DQN) exhibit varying levels of efficiency depending on the population. Notably, the DQN improves accumulated reward over a random recommender by 71.60% for “Foodies”, 65.02% for “Veggies”, 63.46% for “Spanish”, and 8.89% for “Seniors”, while MAB achieves similar performance with fewer resources. Statistically significant differences (*p* < 0.005) are found in the performance of the DQN between populations, with large effect sizes according to Cliff’s delta. These findings highlight recommender systems as an opportunity to navigate market demand, optimize supply chains, and reduce food waste. A better understanding of public preferences enables more effective alignment of supply and demand across the entire food supply chain. As a conclusion, while the DQN effectively captures target group preferences, the optimum recommendation strategy should be chosen by balancing algorithmic performance, computational efficiency, and the specific requirements of the food sector.

## 1. Introduction

The food delivery sector in Europe was valued at USD 55.34 billion in 2022 and is projected to reach USD 69.77 billion by 2028 [1]. This growth is partly driven by the rising demand for balanced diets and wellness programs in senior care centers, schools, and the healthcare sector. To sustain this trajectory, the industry must not only adapt to evolving consumption patterns but also align with broader objectives across the entire food supply chain, such as reducing waste and enhancing consumer appeal [2]. Food delivery services in settings such as schools, senior centers, and hospitals share a common challenge: understanding and meeting consumer preferences [3].

In this context, recommender systems represent an opportunity to navigate market demand. These systems have proven effective at learning consumer preferences, reducing search times, and enhancing the overall user experience. Consequently, they can be found in many different applications, such as podcast reproducing apps [4], app distribution platforms [5], or news sites [6]. When applied to food services, recommender systems can support personalization while also contributing to optimizing supply chains and reducing food waste. By improving the alignment between food offerings and consumer demand, personalized recommendation systems not only enhance consumer satisfaction but also help reduce costs associated with surplus inventory and unsold goods [7]. This alignment allows companies to cut down on waste, improving both economic performance and sustainability [8]. Moreover, the reduction in food waste has an important environmental impact, decreasing the need for resources such as water, energy, and land, which are required to produce, transport, and dispose of unsold food [9]. These benefits contribute to broader goals of sustainability and climate change mitigation [10]. The better we understand consumer preferences, the more effectively we can balance supply and demand.

To adapt to current trends, various types of algorithms have been proposed, ranging from traditional methods, those that use only distance metrics between users or products [11,12] or estimate user preference probabilities [13,14], to more complex approaches that generally rely on deep learning strategies [15,16,17]. Most recommender systems are based on collaborative-filtering techniques [18,19,20,21,22,23,24], which belong to the traditional category. Because most collaborative-filtering approaches rely on numerical scale assessment, the recommendation problem is often framed as a classification task with standard evaluation metrics [25]. Reinforcement learning (RL) offers another compelling approach for recommender systems, and it has been increasingly adopted due to its ability to capture and adapt to user behavior over time [26,27]. Unlike traditional methods, RL can dynamically adjust recommendations based on evolving preferences, enabling more personalized and context-aware suggestions [28].

Previous studies have shown that food choices vary among different cultures [29,30], being influenced by personal situations [31,32] and even by age [33,34,35]. Because of this, different recommendation approaches may be required depending on the characteristics of the target population [36,37,38]. We have not found any conclusive research on recommender systems applied to diverse populations, even though evidence shows that different groups of people respond differently to the same recommendation [39,40]. To the best of our knowledge, the datasets used to evaluate recommender systems vary considerably across studies [41]. In particular, user feedback is often captured through the ratings each user gives to different interactions (purchases, views, etc.), either by using a numerical scale, the most common approach in previous work, or by analyzing user clicks or selection data. To obtain such data, some studies generate their own datasets [42,43,44], while others use publicly available ones [45,46,47,48,49]. In studies where custom datasets are created, participants are usually asked to complete questionnaires, or volunteers simulate interactions based on predefined profiles, which typically results in a limited dataset size. By contrast, publicly available datasets often contain thousands or even millions of user ratings or clicks, which only need to be adapted to the input specifications of the algorithm. These public datasets usually contain anonymized user clicks or ratings collected from various websites or services. However, these users are generally uncharacterized, as personal data are not provided. This lack of information ensures user privacy but prevents comparisons and evaluations across different population groups.

To address this gap, we propose a fuzzy logic-based population simulation, which enables the generation of diverse user groups according to age, gender, geographical area, and city size. Fuzzy logic is particularly well suited for this task due to its adaptability to real-world complexity, and it has already been applied in areas such as control systems [50], robotics [51], and decision-making problems [52]. Previous work has also applied fuzzy logic to simulate populations [53].

The main objective of this study is to demonstrate how the performance of reinforcement learning-based recommender systems for food services varies across different simulated populations, and how these insights can ultimately help optimize supply chains and reduce waste by aligning recommendations more closely with consumer preferences. This study addresses the following research question:

- How do the characteristics of different populations influence the performance of personalized meal recommender systems?

This paper presents the following contributions:

- A fuzzy logic-based approach for generating user profiles that account for diverse populations and varied preferences.
- A methodology for comparing and evaluating reinforcement learning-based recommender systems across different populations.

The paper is structured as follows: In Section 2, the proposed methodological approach is presented. In Section Fuzzy Logic (FL), fuzzy logic (FL) is described to generate a population and four subsets of users. These subsets, or groups, are used to evaluate the recommender systems (RSs) presented in Section 2.3. Section 3 presents the simulation results, divided into two subsections: Section 3.1, which describes the generated populations, and Section 3.2, which discusses the results of the comparison of the recommender systems. Finally, Section 5 presents the conclusions.

## 2. Materials and Methods

To ensure the reproducibility of the study, this section provides a detailed description of the techniques and materials employed. In Section 2.1, the dataset generated for the application is introduced, whereas in Section 2.2, the different modules that compose the proposed methodology are described.

### 2.1. Materials

The dataset was designed to contain a diverse array of dishes, including traditional, innovative, vegetarian, and vegan options, to promote a balanced diet and provide suitable dishes for every simulated population. Using this database, daily menus are generated by selecting random dishes with category tags (*t*): rice, pasta, potato, legume, vegetables, white meat, red meat, fish, fried, egg, dairy, and fruit. Although 12 tags are listed, some of them can represent either first or second courses. To ensure that vegetarian and vegan users have suitable options, at least one vegetarian option is guaranteed in every eligible group of dishes. The database is described in Table 1 and available in the Supplementary Materials.

### 2.2. Methods

**Figure 1:**
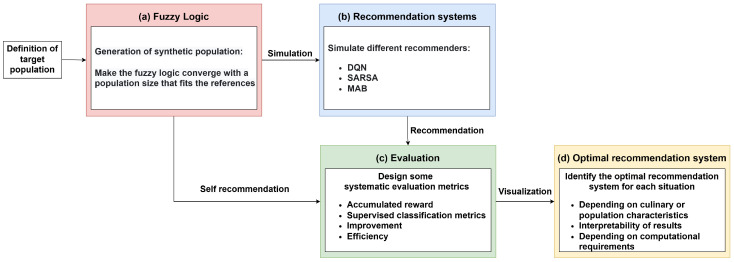
Scheme of the proposed methodology that includes (**a**) the generation of synthetic population, (**b**) simulation of different recommendation systems, (**c**) evaluation of the results, and (**d**) identification of optimum strategy for each case.

The methodology consists of four main modules: fuzzy logic, recommender systems’ simulation, evaluation of results, and selection of the optimum recommender system. Figure 1 illustrates the interconnections between these modules. The fuzzy logic-based module is responsible for generating target populations, which are then used by the recommender systems’ simulation module to obtain results for each simulated group. Once the results are obtained, they are evaluated using the metrics proposed in Section 2.3.3. Finally, based on the evaluation data, the best recommender algorithm is selected.

#### Fuzzy Logic (FL)

For the study, it was necessary to obtain different user profiles with diverse requirements and tastes in order to evaluate how the recommender systems performed in different scenarios. Simulation as an evaluation tool for recommender systems is widely used [54,55], and fuzzy logic [56] is considered an effective approach to meet this need. Therefore, a fuzzy logic tool was developed to simulate different populations. In our case, reference values for user characteristics were extracted from previous studies [57,58,59]. For example, 6% of the population in Spain above the age of 15 is vegetarian or vegan. These variables were used to generate the culinary preferences of different populations in Spain, including age, gender, type of locality (coastal, inland, etc.), and level of preference for innovative recipes. Fuzzy logic took demographic data or user characteristics as input and generated user preferences as output. These preferences were then used to simulate user selection and to train the proposed recommender systems.

**Figure 2:**
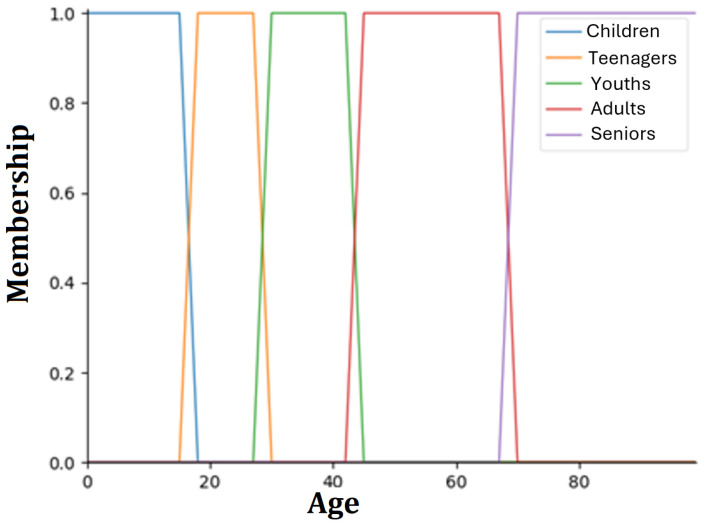
Membership functions for different age categories in a fuzzy logic system. The plot illustrates the membership levels across five age groups: children (0–16 years), teenagers (14–25 years), youths (22–32 years), adults (42–70 years), and seniors (68+ years). The x-axis represents age, while the y-axis indicates the degree of membership, ranging from 0 to 1. Each line represents the fuzzy set corresponding to an age category, showing how membership in these categories changes with age.

**Figure 3:**
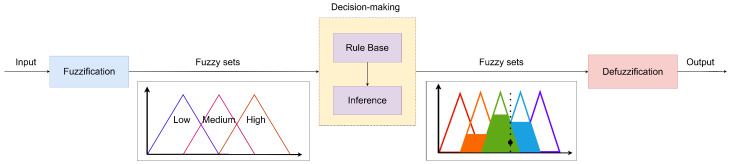
Diagram of the fuzzy logic process. This consists of a fuzzification phase where the input fuzzy sets (corresponding to the input variables) are generated, followed by a decision-making phase in which, based on these input fuzzy sets, the output fuzzy sets (corresponding to the output variable) are calculated, and finally, the defuzzification, where using the output fuzzy set, the output value is calculated.

The first step to apply FL is fuzzification, which involves converting the input values into fuzzy sets. These fuzzy sets may contain elements in partial membership. Each set is associated with a membership function that indicates the degree to which an element belongs to that set (Figure 2). This means that the boundaries between categories are not rigid, allowing elements to partially belong to multiple sets. The slight overlaps between sets reflect this flexibility, enabling a more realistic representation of real-world situations where transitions between categories (e.g., “young” and “middle-aged”) are gradual rather than abrupt. After fuzzification, it is necessary to define the decision-making process based on prior knowledge. The decision-making process is composed of heuristic rules, which consist of the antecedent (the IF part of the rule) and the consequent (the THEN part of the rule). The fuzzy logic inference system consists of three rule blocks. The first block classifies a profile based on its characteristics, such as age, gender, and location, assigning it to one of four diet types: vegan, vegetarian, flexitarian, or omnivore. This block contains a total of 17 rules. The second block generates a set of probabilities to determine the tastes of each profile, using the aforementioned variables and a set of 30 rules. These rules includes combinations of two to eight variables to define user preferences and incorporate multiple conditions. Finally, the third block consists of 15 rules to assess whether the profile tends to be innovative. The information obtained from each of these blocks is combined to create a more complete and detailed profile, enhancing the overall analysis. As a result of the heuristic rules, a final area is obtained, composed of several areas (due to the fuzzy sets) overlapping each other, each corresponding to the result of a rule. Finally, the final area is submitted to the defuzzification process, where the centroid of the total area is calculated, producing the output value. The complete process is shown in Figure 3.

The generated profile is composed of the outputs obtained from the three blocks. The “Diet” variable is obtained from one of these blocks and indicates whether the profile follows an omnivorous, flexitarian, vegetarian, or vegan diet. This is particularly useful because, if the diet is set, for example, to vegan, all animal-derived food tags are assigned a value of 0. The second block of rules sets the tastes of the profile for each of the available labels. For each tag, representing a dish category (pasta, rice, meat, etc.), a percentage is assigned, representing the probability of choosing a dish containing that tag. Finally, the “Innovation” variable is obtained from the third block of rules; this variable indicates the willingness to try new dishes.

The fuzzy logic module provides an individualized profile. This profile is generated from demographic information and is used to extract a set of preferences that reflect the possible consumption habits of the individual. These rules establish relationships between demographic factors and consumption choices. For example, a person who lives in a coastal area tends to have greater access to and predisposition for consuming seafood products [60], while someone living inland may have a greater tendency to consume meat or agricultural products specific to their region. Similarly, factors such as age, gender, and culture influence food selection, establishing patterns that can be simulated by these rules. However, in real life, food preferences do not always follow strict logic based solely on environment or personal characteristics [61]. To reflect this variability and make the generated profiles more realistic, a random factor has been incorporated into the system. This factor allows variations in preferences to be introduced even when demographic conditions suggest another trend. For example, even if a person lives inland, they might develop a strong preference for fish due to personal experiences, family habits, or individual tastes. In this way, the system not only assigns preferences based on predefined rules but also introduces a layer of variability that simulates the actual diversity of food choices in different contexts. This enables more complete and representative profiles to be obtained.

### 2.3. Recommendation Systems (RSs)

This section presents the reinforcement learning (RL) problem considered to provide a personalized menu recommendation system. This study combined the historical selections of the user, menu options, and user choices for model training. Section 2.3.1 presents the menu recommendation problem formulation, whereas Section 2.3.2 describes the algorithms considered for the recommendation system.

#### 2.3.1. Problem Formulation

In this paper, different recommendation systems were explored for menu recommendation. For each course (i.e., first, second, and dessert), the user selected among at least two options, with the system recommending the plate type or tag *t* with the highest estimated value to the user. The recommendation system took as input the previous selections of the user for each course, denoted as $s_{m}$. Formally, the menu recommendation system was modeled as a Markov Decision Process (MDP), defined by the tuple:

(1) $$M=\left(S,A,P,R,\gamma \right)$$

where

- State Space S: A state $s_{m}\in S$ is defined as the historical selection of the user for each course over the past *w* days, up to day *m*. Each day consists of a vector of size three (corresponding to the first course, second course, and dessert) containing the selected plate type (tag) for each course. For instance, a menu consisting of pasta, meat, and fruit would correspond to tag IDs X,Y,Z, respectively.
- Action Space A: The action $a_{m}\in A$ consists of a pair (c,t), where c∈{1,2,3} corresponds to the course index (first, second, or dessert), and *t* is the candidate tag (e.g., pasta) to be recommended. The action space at time *m* comprises all available plate types for selection. Note that not all the tags are available for each course. For example, for the dessert, only fruit and dairy are available. The size of the action space was 15 and the proposed algorithms considered all the tags.
- Transition Probability P: The transition probability $P(s_{m+1}|s_{m},a_{m})$ defines the likelihood of moving from state $s_{m}$ to state $s_{m+1}$ after taking action $a_{m}$. The definition depends on the learning framework:
  – In multi-armed bandit (MAB) settings, the transitions are independent across time steps, as each recommendation does not influence future states.
  – In Q-learning and State–Action–Reward–State–Action (SARSA) settings, the transitions account for sequential dependencies, where the user’s historical choices influence future selections.
- Reward R: Once the user selects a plate for each course, the chosen items are compared with the recommended tags. If the recommendation matches the user’s selection, the system receives a reward $R(s_{m},a_{m})$. This mechanism allows learning without explicit user feedback.
- Discount Factor γ: The discount factor γ∈[0,1] controls the importance of future rewards when selecting the current action. A value of γ=0 leads to a greedy selection based only on immediate rewards, whereas γ>0 incorporates future outcomes into the decision-making process.

These general definitions describe the menu recommendation problem. Specific algorithmic details, including implementation variations, are presented in Section 2.3.2. In summary, the proposed menu recommendation system aims to optimize future rewards by recommending plate types (tags) that align with user preferences.

#### 2.3.2. Algorithms

In this section, different RSs are explored with well-known reinforcement learning-based algorithms [62] to learn user preferences: MAB [63,64], SARSA [65], and Deep-Q Network (DQN) [66]. These methods were selected in this study due to their established presence in the RL literature [13,67] and their frequent application in diverse domains [68,69], providing a strong basis for the comparative analysis of their performance in the context of personalized food recommendations. These methods take the daily menu options as input and provide as output the option with the highest expected accumulated reward, which is then compared with the user selections. User interactions are required to improve performance over time. A prediction is generated for each possible tag, and the one with the highest output value ($Q(s_{m},a_{m})$) is recommended to the user.

**Figure 4:**
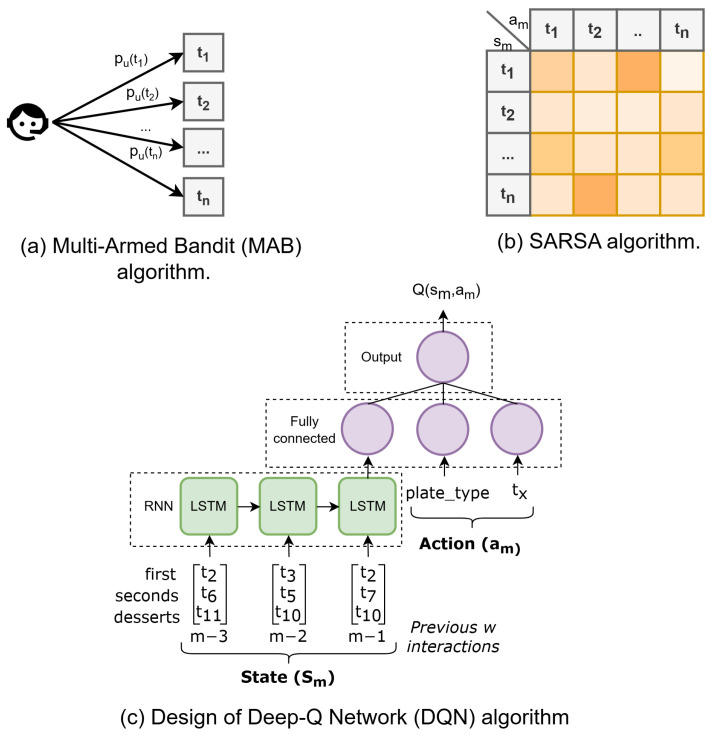
Architectures of the different recommender systems employed in this study, having in common the selection of a given dish ($t_{x}$) for recommendation: (**a**) multi-armed bandit, (**b**) SARSA, and (**c**) Deep-Q Network.

The MAB algorithm (see Figure 4a) chooses the dish to recommend ($t_{i}$) based on learned probabilities ($p_{i}$) for each dish *i*. An epsilon-greedy approach was employed for exploration–exploitation. This strategy balances exploration and exploitation by selecting a random dish with probability ϵ, which, after an optimization process, was set to 0.15. Probabilities are updated by comparing the last dish chosen by the user with the recommendation, where a match results in an increase in $p_{i}$. Other exploration–exploitation strategies were evaluated, with the epsilon-greedy method demonstrating superior performance.

SARSA (see Figure 4b) is based on a table that is updated after each iteration, where the state ($s_{m}$) at moment *m* is defined as the last dish tag (*t*) selected by the user. The action ($a_{m}$) at moment *m* is the recommended dish among the list of available dishes for a given plate type (i.e., first, second, or dessert). Thus, the table used in this algorithm represents the last dish selected by the user (rows) and the expected accumulated reward after recommending any of the available dishes (columns).

The proposed DQN (see Figure 4c) takes as state ($s_{m}$) the selected dish tags for the last *d* days, organized by plate type (i.e., first, second, and dessert), and the action ($a_{m}$) is a pair consisting of the plate type and the tag (*t*) under consideration. The state ($s_{m}$) is the input to a recurrent neural network (RNN) that learns long-term temporal dependencies. The output of the RNN and the action ($a_{m}$) are merged and fed into a deep neural network (DNN) that produces the Q value as output.

The RNN is composed of Long Short-Term Memory (LSTM) [70] cells that are capable of capturing information across multiple time steps. The first time step (i.e., day) is input into the first LSTM cell, and the output is passed to the next LSTM cell so that the second time step can be processed. This procedure is repeated for all time steps (i.e., w−1, where *w* is the number of previous days considered) until the final output is obtained. These cells contain several neurons, and all cells within the same layer share the same parameters. Finally, a feed-forward layer [71] is added to match the output with the number of activities (*t*).

Moreover, for the DQN, which is composed of an RNN and a neural network, an additional set of parameters had to be specified, such as the optimizer function, which defines how the parameters are updated; the number of epochs (number of passes through the entire dataset); and the learning rate (parameter that controls the size of the steps taken during the optimization process to minimize the loss function, thereby affecting how quickly the model adapts to the data). Other parameters, such as the reward (which determines the feedback given to the model based on actions) and the decay factor (which gradually reduces the learning rate over time) were also optimized. In addition, for the Adam optimizer [72], $\beta_{1}$ and $\beta_{2}$ must be set; these are the exponential decay rates for the first- and second-moment estimates, respectively. Finally, the batch size was also tuned, defining the number of instances introduced into the network at each step within an epoch.

For each combination of hyperparameters in the grid search, the DQN was trained and evaluated using several repeated executions of the simulation. The total accumulated reward obtained across these runs was averaged and used to select the best-performing configuration. This repetition allowed assessing the robustness of the model under varying simulated user interactions, serving a similar role to a validation phase by ensuring that the selected hyperparameters generalized well across different simulated scenarios. In addition, dropout was included as a tunable hyperparameter, effectively reducing overfitting and improving the final average accumulated reward. The grid search was conducted jointly across all populations to identify a single set of hyperparameters that provided robust performance and ensured model generalizability. The final configuration used for all experiments is summarized in Table 2, which reports the optimal DQN hyperparameters selected based on the average accumulated reward across all simulated populations.

In order to adapt the RNN to a reinforcement learning approach, several adjustments were made to the training process. First, a target model was maintained, which was a copy of the current model used to compute the Q-value of the next state. This model was updated after a certain number of training steps of the current model. A random factor was also included to balance exploration and exploitation, allowing a random recommendation at a specified rate. Each time the model provided a recommendation and the user selected a dish, the agent’s memory was updated with this information. The model was then trained after a given number of interactions, by randomly sampling past interactions from memory and replaying these recommendations. Through this process, the parameters of the neural network were updated.

For the DQN model, a set of parameters was optimized using a grid search to enhance performance. The hyperparameters adjusted included the window size for previous selections (2–20 days), the number of neurons in the RNN and feed-forward layers (2–128), the batch size (32–128), the retraining frequency (2–10 days), the number of training samples (10–30), the learning rate (0.0001–0.001), the target update interval (2–5), the positive reward range (1–10), the negative reward range (−5–0), the initial training day (10–40), and the decay factor (0.75–0.995). The Adam optimizer was used.

#### 2.3.3. Evaluation

Once the recommendation models were simulated, evaluating the results was crucial to determine the most suitable algorithm for each scenario. To obtain the evaluation metrics, the results were compared with the selections made by each user. For the evaluation of the different RSs, the metrics used included accumulated reward, improvement, efficiency, and supervised classification metrics:

- Accumulated reward refers to the mean of the sum of rewards obtained by each algorithm in the evaluated population. If the recommendation matches the user selection, a reward is provided to the algorithm. This metric indicates how often the algorithm succeeded in recommending the correct item compared with the actual selections.
- Improvement shows the relative increase in performance with respect to a random recommender. To correctly compute this metric, a random baseline is required. For this purpose, the algorithm randomly selected one dish from the three options for the first and second courses, and one from the two options offered for dessert, without considering any probabilities.
- Efficiency measures how often per day the algorithm correctly predicts each selection of the user. In the context of our study, this metric indicated how many dishes, on average, the recommender correctly guessed out of the three choices (first dish, second dish, and dessert).
- Supervised classification metrics: this includes F1-score, recall, and precision [25], which are commonly used in supervised classification. These metrics assess the effectiveness of recommendations based on user-selected items. A value of 1 indicates perfect recommendation performance (i.e., the system recommends exactly the same items chosen by the user), while 0 represents the lowest performance.

Apart from these metrics, the selections of the algorithms are depicted along with the Optimum, which represents the probabilities of user preferences, and the random algorithm, which provides the baseline for benchmarking. To compute the Optimum, the probability array associated with each user was used. When characterizing each population, user characteristics were obtained, including the probability of choosing each category tag. Using these probabilities, user selections were simulated, introducing variability in preferences, as users may choose different dishes on different days, even if they have a favorite. The entire evaluation process of the different algorithms is summarized in Algorithm 1.    
**Algorithm 1:** Menu recommendation process for all algorithms
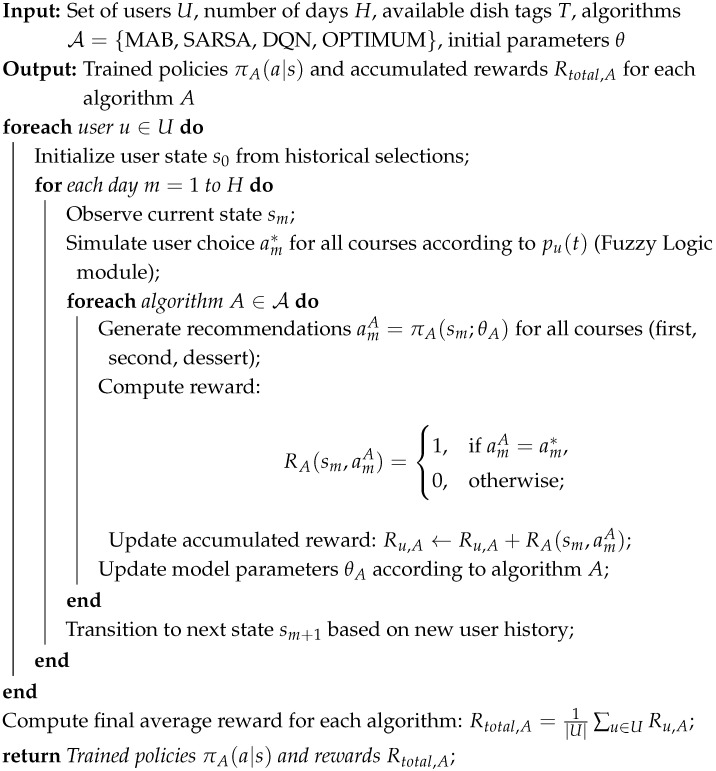


#### 2.3.4. Optimal Recommendation System Selection

In this final module, once all the previous modules have been executed, the optimal recommendation system is selected. For this selection, different factors are considered: the results of the evaluation metrics and the visualizations plotted (see Section 2.3.3), the intended purpose of the algorithm, and the computational requirements. All these factors inform and guide the decision toward selecting the most suitable algorithm for each scenario.

## 3. Results

In this section, the results of applying the methods explained in Section 2 are presented. In Section 3.1, a population is generated, and four target groups are characterized, whereas in Section 3.2, the results of training the proposed RSs are depicted for each group.

### 3.1. Characterization of Different Target Populations

**Figure 5:**
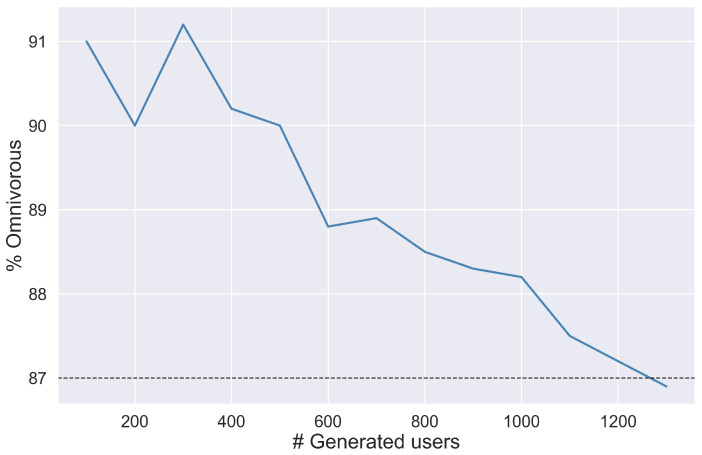
The percentage of omnivores in the population as the number of generated users increases. The black line indicates the reference value [73]. The symbol # denotes the number of items.

In this experiment, a population of users was generated using the fuzzy algorithm outlined in Section 2, from a set of meaningful variables (i.e., age, gender, etc.). The objective of this experiment was to obtain a representative population of users whose characteristics were close to those found in the literature about the Spanish population [73]. The number of users to be generated is presented in Figure 5, and Table 3 shows the attributes of the selected four target groups (50 users per group).

Figure 5 shows how the reference value is approximated (87% of omnivores in the Spanish population) as more users are generated, with oscillations due to the randomness of the generation process. It is important to note that this percentage was not used as a fixed threshold but rather as a reference value to validate the simulation process. The results illustrate how the generated population progressively converged towards this reference value as the number of users increased, confirming the correct calibration of the fuzzy generation model. This was performed with the rest of the variables (i.e., percentage of veggies, females, etc.). In conclusion, the proposed fuzzy engine was correctly adjusted to approximate the reference values of the Spanish population when generating 1300 users.

Finally, from this Spanish population, four groups were chosen for the evaluation (see Table 3). These four subsets represented the population of Spain considering the entire population, older adults (“Senior”), “Foodies”, and “Veggies”.

### 3.2. Recommendation Systems (RSs)

In these sets of experiments, each RS was trained and evaluated with a simulation process that took as input the groups of users generated in the previous step (see Section 3.1). The objective of these sets of experiments was to propose efficient algorithms for the delivery of personalized recommendations and to understand how the different algorithms behaved among populations with different characteristics and preferences. Note that both the “Foodie” and “Veggie” populations possessed distinct tastes, and the vegetarian group experienced a more limited range of choice options.

**Figure 6:**
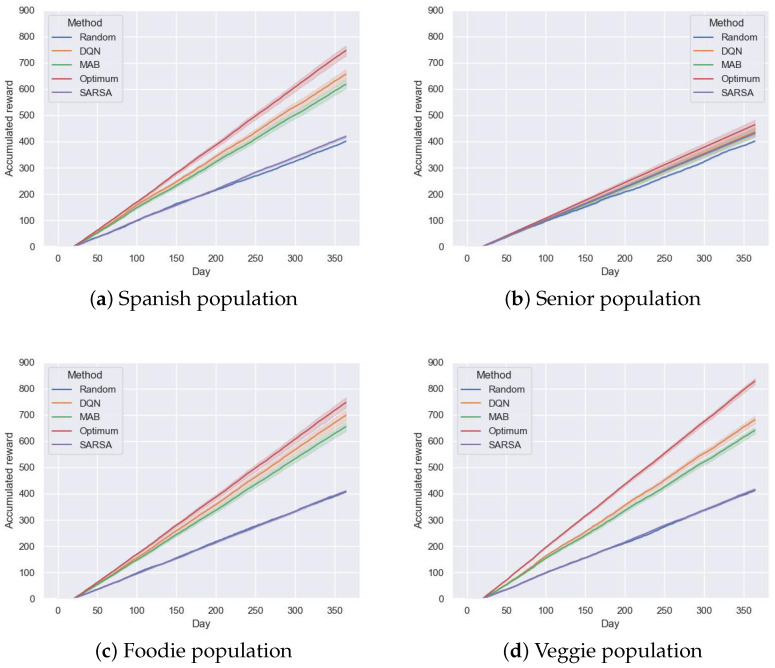
Graph plotting the performance of the algorithms against different populations. For each day and user, three recommendations were computed (one per plate type), and one was summed for each if the selected plate matched the recommendation.

After carrying out preliminary research, both the MAB and the SARSA algorithms delivered random recommendations with a probability of 15% to balance between exploration and exploitation. The DQN algorithm used the selections of the last three days (d = 3) and the likelihood of delivering a random recommendation was 15%, which decreased by 1% after each training to a minimum of 1%. For the recurrent neural network (RNN), LSTM blocks had 32 neurons, and the following deep neural network (DNN) had one layer of 16 and an output layer of 1 neuron. The learning rate was set to 0.0005, the target network was updated after three training processes, and the algorithm trained after every 20 recommendations. The random seed was set to 10, and the training process involved 15,000 training episodes. The results of the different algorithms over 365 simulated days are presented for each group in Table 4 and Figure 6. Training involved providing recommendations and comparing them with user selections over 20 days. The DQN was then retrained using a subset of data from the replay memory, and this process repeated continuously. Both MAB and SARSA were updated daily.

Table 4 shows that the DQN outperformed the SARSA and MAB algorithms, achieving the best performance compared to the random algorithm. The highest improvement corresponds to the “Foodie” group with 71.60%, followed by the “Veggie” group with 65.02%, with an improvement of 63.46% and 8.89% for the “Spanish” and “Senior” groups, respectively. Note that the “Senior” group had the one with the lowest number of innovative users (Table 3). In this case, the result achieved by SARSA for the “Senior” group showed an improvement of 7.59% compared to the random algorithm, indicating that this method worked better than MAB when user preferences were not so clearly defined. Finally, in terms of efficiency, on average, the DQN correctly recommended two dishes out of three.

A comparison across different populations showed that the best efficiency was achieved for the “Foodie” group, which also attained the best F1 score and recall. This group exhibited a mean efficiency of two out of three dishes correctly recommended (Table 4), an F1 score of 0.6754, a recall of 0.6722, and a standard deviation of the accumulated reward of 120.22. The “Veggie” population demonstrated similar performance, with a maximum efficiency of 1.97, an F1 score of 0.6673, a recall of 0.6576, a precision of 0.6894, and a standard deviation of the accumulated reward of 83.60. However, that population had a difference in the accumulated reward with respect to the Optimum of more than 125 points. Regarding the “Spanish” group, although the accumulated reward was lower with a score of 655.74, the improvement with respect to the random algorithm was 63.46%, with a standard deviation of 118.05. The F1 score, recall, and precision for this group were 0.6448, 0.6371, and 0.6664, respectively. Finally, the “Senior” population obtained a maximum efficiency of 1.27 with an improvement with respect to the random algorithm of 8.89%, and a standard deviation of 110.65. The corresponding F1 score, recall, and precision were 0.4198, 0.4216, and 0.4315. These results suggest that RSs have less added value in the “Senior” population.

Figure 6 depicts a comparison between the proposed algorithms over one year considering one meal per day, composed of a first plate, second plate, and dessert. The random method indicates a baseline, expecting any other algorithm to achieve better performance, and the Optimum represents the highest achievable accumulated reward (see Section 2.3.3).

After day 100, the distance between the performance of MAB and the DQN became more apparent for the “Spanish”, “Foodies”, and “Veggies” groups. The SARSA algorithm obtained the lowest results presenting (at the four cases) very similar performance to the random method. That might be due to the table behind this algorithm that required a higher number of interactions. This can be seen in Figure 6. For example, in the case of the “Spanish” population, SARSA achieved an improvement of nearly 4.41% with respect to the random method. Analyzing the results, all three proposed recommendation systems showed worse performance than the Optimum, though most of the time, they performed better than the random method. This is because the Optimum shows the maximum possible improvement of the RSs, calculated by directly using the percentages of choosing each food group tag from the calculated profiles. As these preferences are probabilities, even the Optimum does not reach the accuracy to correctly recommend three out of three dishes. In this sense, it seems clear that the more determined the profile of the users in a population, the steeper the slope. For example, the “Senior” population did not have a clear preference for certain dishes, reaching the Optimum with an accumulated value of 464, followed by the population of “Spanish” eaters with an accumulated reward of 746. Finally, due to the choice (offer) limitations, the populations of “Foodies” and “Veggies” had a significantly higher cumulative score, achieving 747 and 828, respectively.

**Figure 7:**
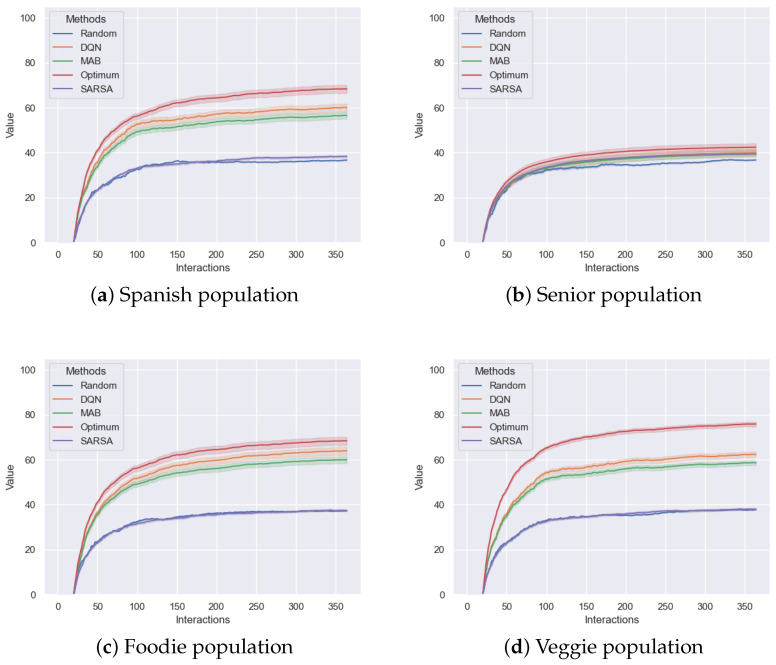
Comparison of the different methods showing the percentage of accurate recommendations relative to the total recommendations.

Figure 7 presents the percentage of accurate recommendations relative to the total number of recommendations. The proposed profiles do not exhibit temporal evolution, and as a result, the figure clearly indicates that, within a year, the algorithms reached a stable performance with no signs of further improvement.

Table 5 presents *p*-values and Cliff’s delta for pairwise comparisons of the DQN algorithm. In contrast, Table 6 reports the corresponding values for the differences between Optimum and DQN outcomes (i.e., performance gap), highlighting the deviations of each population from the optimum.

When comparing the four populations (“Spanish”, “Foodie”, “Senior”, “Veggie”), significant differences were seen in both absolute DQN values and performance gaps (p<0.005). Notable examples of statistically significant differences in the performance of the DQN between populations include Spanish–Senior ($p=9.1\times 10^{-7}$, δ=0.65; gap: $p=2.4\times 10^{-13}$, δ=0.97), Foodie–Senior ($p=2.3\times 10^{-7}$, δ=0.67; gap: p=0.86, δ≈−0.02), and Senior–Veggie ($p=1.5\times 10^{-7}$, δ=−0.70; gap: $p=4.0\times 10^{-13}$, δ=0.97). Only Spanish–Veggie showed no significant differences (absolute: p=0.75, δ≈−0.04; gap: p=0.87, δ≈−0.02). These results indicate that the populations behaved differently both in achieved performance and in deviation from the Optimum, supporting the use of population-specific reinforcement learning strategies.

As a conclusion, the DQN proved to be an effective method to model user preferences. It was also observed that the maximum achievable performance of that method was conditioned by the selections of the users (i.e., Optimum), whether they corresponded to a population with clear preferences (i.e., “Foodie” group) or to a population with a higher level of uncertainty when making a selection (i.e., “Senior”).

## 4. Discussion

This paper presented an evaluation framework to assess the performance of a set of recommendation systems without the need for explicit user feedback. For that, a simulation process was designed to generate user profiles with certain preferences using a fuzzy logic approach. This method allowed the selection of a dish given the traditional daily options available in the food domain. Our results exhibited the difficulties of recommending to a population (in this case Spanish population) that is mainly omnivorous (87% in this case) and with very inconsistent preferences. Such heterogeneity not only challenges algorithmic performance but also mirrors the difficulties faced by the food supply chain: when consumer demand is highly unpredictable, it becomes harder to optimize supply, reduce waste, and design offers that remain attractive to consumers. Moreover, we showed the Optimum representing the maximum achievable performance, with no room for improvement in the proposed RS algorithms in this population and simulation scenario. Statistical analysis of the results, including *p*-values and Cliff’s delta, showed that the four populations differed significantly from each other (*p* < 0.005) in both absolute performance and deviation from the Optimum, confirming that distinct behavioral patterns exist and justifying population-specific modeling strategies.

In terms of performance, although the DQN achieved the highest scores, the MAB algorithm performed reasonably well. There was only a maximum difference of 0.12 in terms of efficiency with respect to MAB for the “Foodie” population. A more detailed comparison of both RSs shows that the DQN is a much more complex algorithm that can handle more information. With this additional information, it can generalize to all users, but it loses interpretability and requires more computational resources. Although MAB is more interpretable as it relies on a set of probabilities, the fact that the DQN can incorporate an increasing number of variables allows an inspection of the utility of different factors that may contribute (or not) to provide more accurate recommendations. In addition, the standard deviation of the accumulated reward highlighted differences in consistency across algorithms: the DQN often showed higher variability than MAB, indicating that while it can achieve higher rewards, the outcomes can fluctuate more across users or days, whereas MAB tends to provide more stable results. However, as seen in Figure 6, as the number of days increased (i.e., interaction data), the improvement of the DQN with respect to MAB increased. These results indicate that although DQN was the best-performing RS, the benefits were more noticeable with a high number of user interactions for those groups with clearer preferences (i.e., “Foodies”, “Spanish”, and “Veggies”). This pattern was consistent with the observed standard deviations: groups with higher accumulated rewards generally exhibited slightly lower variability, suggesting more consistent user preferences, while groups with lower rewards showed larger fluctuations. Thus, the selection of the algorithm depends on the needs of the researcher or enterprise, the DQN being of interest if there are variables that require studying as their influence in the selection made by the user is unknown. The results also showed that in those groups with a higher accumulated reward, the dish preferences were more consistent, which in a real-world setting could translate into better alignment between demand forecasting and supply-chain decisions.

Following this analysis, the results support the theoretical assumption that the specificity of a population’s culinary preferences directly influences the complexity of recommendation tasks. Populations with strong, narrowly defined preferences are empirically easier to model and provide recommendations for, whereas populations with broad, uniform consumption patterns present greater challenges for all algorithms, confirming the expectation that preference dispersion increases modeling difficulty. Similarly, the observed differences in offer constraints, where populations with limited options (e.g., “Veggies”) are easier to model than those with a wide variety, highlight the practical relevance of aligning recommender complexity with the characteristics of the target population. If the target population falls into a group with a very wide offer and low tendencies in culinary preferences, more complex recommender systems should be used and vice versa. Beyond algorithmic considerations, this reasoning has implications for marketing and supply chain optimization: populations with clearer preferences make it easier to design attractive offers, forecast demand, and minimize waste, while diverse populations require more adaptive strategies to balance variety with efficiency. In practical terms, these insights could guide the implementation of recommendation systems in real contexts such as restaurants, school canteens, or catering services, where understanding population-specific tendencies can improve menu planning, reduce operational costs, and enhance user satisfaction. Furthermore, the proposed framework could serve as a decision-support tool for the hospitality and food industry by helping managers align culinary offerings with both user demand and logistical constraints.

Some limitations of the proposed methodology might be related to the preferences of the users generated by the fuzzy logic. This approach was adjusted so the output matched the reference values of different culinary groups for a given population such as the proportion of omnivores in the Spanish population. However, more specific variables considered in this study were not found in the literature, like the preferences for some tags such as pasta or meat, for different ages, or gender. Note also that these tags may vary across studies, as there is no consensus on the specific tags used to categorize different dishes. These tags may also vary in relation to the territory, traditions, and current tendencies, among others. In addition, user preferences were assumed to remain stable throughout the year, although in practice they may change over time due to factors such as seasonality, weather conditions, or variations in the health status of the user. Moreover, the current menu-generation process does not explicitly consider logistical or seasonal constraints such as ingredient availability or cost, which should be acknowledged as an additional limitation of the current setup. On the positive side, the proposed methodology is flexible enough to allow researchers or practitioners to introduce very different populations with their own tastes and preferences, making it adaptable to a wide variety of contexts. In addition, it should be understood as a holistic approach that needs to be readjusted for each use case considering very different factors such as the characteristics of the target population (being served), the availability of data for scoring the choices, or the computational resources.

A review of the state of the art shows the added value of the proposed methodology for existing work. For example, the authors of [74] identified several challenges in previous research on RSs, including privacy concerns, data collection constraints, and the need for accurate and representative information. Additionally, they mention the complexity of selecting the appropriate implementation and the narrow focus on accuracy in evaluating RSs. Similarly, references [75,76] noted that earlier works relied on datasets specific to one region or limited to particular systems and that many studies only considered a limited number of attributes for providing the recommendations. This paper addressed those limitations by proposing a methodology that simulates interactions through the selection of characteristics of the target population, avoiding privacy issues. It also systematically evaluated various algorithms and configurations within the same target population to identify the most effective solutions and utilized a diverse set of metrics, providing a more holistic measure of system effectiveness. Moreover, it enabled the generation of populations based on demographic data independent of regional constraints and allowed the inclusion of numerous attributes involved in the generation. These aspects bring opportunities not only for advancing RS research but also for applying these systems in the food sector to anticipate consumer demand, support marketing strategies, and contribute to a more efficient and sustainable food supply chain.

The findings of this study offer valuable insights for improving market segmentation, supply planning, and demand forecasting. The differences in algorithmic performance across population segments suggest that tailoring recommendation systems to specific user groups can enhance both customer satisfaction and business efficiency. For populations with clearer preferences, such as “Foodies” or “Veggies”, more personalized recommendations can optimize product offerings, improving demand forecasting and reducing waste. Conversely, more diverse populations may require adaptive algorithms that account for a wider range of preferences. These insights could help businesses target marketing efforts more effectively and align supply chains with actual demand, fostering sustainability by minimizing food waste and optimizing resource use. In practical terms, the results can support data-driven decision-making for stock and production planning, improve demand forecasting accuracy, and inform the design of sustainable nutrition policies by aligning menu offerings with both consumer preferences and environmental objectives. Furthermore, these models could be integrated into digital food platforms such as online canteens, delivery services, or menu-planning applications to dynamically adjust meal options based on predicted user preferences and ingredient availability. By continuously matching recommendations with real-time data on demand and stock levels, such systems could help kitchens or suppliers plan production more accurately, minimize surplus, and reduce food waste while keeping menus aligned with consumer interests. The proposed framework could be implemented in institutional food services such as hospitals, elderly care centers, or school meal programs to dynamically adapt menus to user profiles and ingredient availability, improving satisfaction and minimizing waste, while user interaction is modeled without requiring personal data, relying only on user identifiers and historical selection records.

This methodology significantly improves the flexibility and accuracy of RSs by addressing key challenges such as regional dataset limitations and the inclusion of multiple attributes. However, it is important to consider certain drawbacks and areas for improvement. For example, user preferences are currently assumed to remain stable throughout the year, whereas in practice they may fluctuate due to seasonality, weather, or changes in the health status of the user. Additionally, the current menu-generation process does not explicitly account for logistical or seasonal constraints such as ingredient availability or cost. Whereas the simulation of interactions based on demographic data provides a robust alternative to traditional data collection methods, it may not fully capture the complexity of real-world user behavior. Additionally, the process of selecting and weighting numerous attributes could introduce biases if not carefully managed. Future research should focus on refining these simulations and developing more sophisticated techniques for attribute selection to further enhance the reliability and applicability of recommender systems in the food domain, as well as their capacity to optimize supply chains and reduce food waste through a better understanding of consumer preferences. Moreover, integrating psychographic and cultural factors could provide a more detailed understanding of user preferences, and testing the model in real-world settings would help validate and refine its practical applicability.

Table 7 summarizes the characteristics, advantages, and limitations of datasets used in previous recommender system studies. This comparison highlights the limitations of traditional approaches, such as reliance on homogeneous or anonymous datasets, and contrasts them with the methodology proposed in this paper, which allows for a more comprehensive evaluation by incorporating diverse populations.

## 5. Conclusions

Our findings revealed significant variations in the performance of recommender systems based on the characteristics of the population. On the one hand, we simulated menu selection by individual agents characterized by a fuzzy engine. On the other hand, for a given population, we assessed which recommender system was more suitable. In this way, reference statistics were used from previous works to characterize a population of 1300 users that could very well approximate a European region with a certain culinary tradition. This population was divided into four subgroups to evaluate the suitability of recommender systems (RSs) in different settings: “Spanish”, “Foodies”, “Veggies”, and “Senior”. These subgroups followed the entire Spanish demographics, senior population in Spain, vegetarian communities, and the so-called Foodies. These strata represented different parameters in terms of having clear preferences or not, having access to a wider offer or not, choosing very different options or not, among others. Statistical analysis using *p*-values and Cliff’s delta confirmed that these subgroups differed significantly both in achieved performance and in deviation from the Optimum, highlighting the importance of population-specific recommendation strategies.

The results showed that fuzzy logic could be used to approximate the statistics of a certain region when considering demographic data, the culinary tradition, the catering offer, etc. By focusing on these characteristics, which we considered most relevant to defining the populations, the study highlighted significant differences in the performance of recommendation algorithms. Moreover, the methodology is adaptable to any set of labels, enabling its application in various contexts beyond the chosen subgroups. Three different recommendation algorithms were evaluated over these groups of users: SARSA, MAB, and DQN. The results showed that the DQN achieved the best recommendation performance compared to SARSA and MAB with a maximum efficiency (i.e., mean number of dishes selected by the user that have been recommended) of two out of three for “Foodies”. However, for the “Senior” group, the efficiency was similar for the three RSs, achieving a value around 1.25, with a maximum improvement compared to the random RS of 8.89%.

In conclusion, the selection of the recommendation method depends on the specific use-case characteristics, including the requirements in terms of interpretability, the computational cost, or the characteristics of the target population. Beyond individual personalization, recommender systems also represent an opportunity to better understand consumer preferences, making products more attractive, supporting marketing strategies, and ultimately helping to navigate market demand. As such, they can play a role in optimizing supply chains and reducing food waste across the entire food supply chain. Future research could focus on validating the fuzzy logic model with real user data from a variety of demographic groups. Comparing these actual user profiles with the simulated ones would allow us to refine the model, improving its accuracy and applicability in real-world settings. Moreover, to extend the analysis to include a wider variety of algorithms, testing their adaptability and effectiveness in different demographic contexts will help determine whether certain approaches can be optimized for specific populations, leading to more personalized, accurate, and sustainable recommendations.

## Figures and Tables

**Table 1:** Description of the database. This table provides an overview of the contents of the database, including the distribution of plate types, the number of plates in each category, the average number of tags associated with each plate type, and the count of innovative dishes. Finally, the “Example” column presents sample dishes from each category, offering a glimpse into the variety and complexity of the dishes contained in the database. The symbol # denotes the number of items.

Plate Type	Count	#Tags	#Innovative	Example
Firsts	60	5	10	Rice with tomato, pumpkin risotto
Seconds	60	8	23	Zucchini omelet, sweet and sour pork
Desserts	25	2	4	Cheesecake, mango

**Table 2:** Configuration parameters for the recommendation algorithms.

Parameter	MAB	SARSA	DQN
Random recommendation probability	15%	15%	15%, decreasing by 1% per training (min 1%)
Exploration–exploitation balance	ϵ-greedy	ϵ-greedy	ϵ-greedy
Previous selections used (*d*)	–	–	3 days
LSTM neurons	–	–	32
DNN structure	–	–	1 hidden layer (16 neurons) 1 output layer
Learning rate	–	–	0.0005
Target network update	–	–	Every 3 training runs
Training frequency	Real time	Real time	Every 20 recommendations
Training duration	20 days	20 days	20 days

**Table 3:** Characteristics of the subsets analyzed containing information about the percentage of females, the age ranges, the diet type, and the percentage of innovative users. The diet indicates the percentage of omnivores, flexitarians, vegetarians, and vegans, respectively. 1* Age range according to Spanish demographic curves.

Group	% Female	Age Range	% Omnivorous	% Flexitarians	% Vegetarians	% Vegans	% Innovative Users
Spanish	52%	1*	80%	16%	2%	2%	45%
Foodies	44%	Any age	72%	20%	6%	2%	88%
Veggies	70%	Any age	0%	50%	30%	20%	88%
Senior	54%	>70	98%	2%	0%	0%	10%

**Table 4:** Evaluation metrics obtained for each recommendation system and group after a simulation of 365 days. Improvement is the increase in percentage of the accumulated reward compared to the random algorithm, whereas efficiency shows the mean number of correctly recommended dishes out of 3. The best results are marked in bold.

Recommendation System	Group	Accumulated Reward	Std. Dev.	Improvement	Efficiency	F1	Recall	Precision
DQN	Spanish	655.74	118.05	63.46%	1.9	0.6448	0.6371	0.6664
	Foodie	**698.08**	120.22	**71.60%**	**2.02**	**0.6754**	**0.6722**	0.6835
	Veggie	680.72	**83.60**	65.02%	1.97	0.6673	0.6576	**0.6894**
	Senior	436.91	110.65	8.89%	1.27	0.4198	0.4216	0.4315
MAB	Spanish	617.44	101.37	53.91%	1.79	0.6058	0.5998	0.6220
	Foodie	**654.85**	102.47	**66.97%**	**1.90**	**0.6339**	**0.6335**	0.6370
	Veggie	640.50	**72.26**	55.26%	1.86	0.6273	0.6216	**0.6413**
	Senior	431.23	95.01	7.48%	1.25	0.4160	0.4180	0.4242
SARSA	Spanish	418.86	36.99	4.41%	1.21	0.3837	0.4088	0.4042
	Foodie	408.78	30.32	0.48%	1.18	0.3846	0.3966	0.4033
	Veggie	415.25	33.04	0.65%	1.20	0.3914	0.4043	0.4018
	Senior	**431.69**	**30.24**	**7.59%**	**1.25**	**0.4032**	**0.4175**	**0.4094**

**Table 5:** Results of statistical tests for pairwise comparisons reported with *p*-value and Cliff’s delta for the DQN algorithm.

	Spanish	Foodie	Senior	Veggie
Spanish	*p* = 1 δ=0	*p* < 0.005 δ=−0.47	*p* < 0.005 δ=0.65	*p* = 0.75 δ=−0.044
Foodie	*p* < 0.005 δ=0.47	*p* = 1 δ=0	*p* < 0.005 δ=0.67	*p* < 0.005 δ=0.44
Senior	*p* < 0.005 δ=−0.65	*p* < 0.005 δ=−0.67	*p* = 1 δ=0	*p* < 0.005 δ=−0.7
Veggie	*p* = 0.75 δ=0.044	*p* < 0.005 δ=−0.44	*p* < 0.005 δ=0.7	*p* = 1 δ=0

**Table 6:** Results of statistical tests for pairwise comparisons reported with *p*-value and Cliff’s delta for the difference between the DQN algorithm and the Optimum.

	Spanish	Foodie	Senior	Veggie
Spanish	p = 1 δ=0	*p* < 0.005 δ=0.7	*p* < 0.005 δ=0.97	*p* = 0.87 δ=−0.023
Foodie	*p* = *p* < 0.005 δ=−0.7	*p* = 1 δ=0	*p* = 0.86 δ=−0.024	*p* < 0.005 δ=−0.7
Senior	*p* < 0.005 δ=−0.97	*p* = 0.86 δ=0.024	*p* = 1 δ=0	*p* < 0.005 δ=−0.97
Veggie	*p* = 0.87 δ=0.023	*p* < 0.005 δ=0.7	*p* < 0.005 δ=0.97	*p* = 1 δ=0

**Table 7:** Summary of dataset characteristics, advantages, and limitations in previous recommender system studies.

Type of Study/References	Dataset Characteristics and Advantages	Main Limitations
Studies using public datasets	Contain large-scale real user interactions (clicks, ratings, purchases, etc.).	Users are anonymized and lack demographic, cultural, or contextual information.
(e.g., Wu et al. [45], Harper and Konstan [46],	Facilitate benchmarking and reproducibility across different algorithms.	Prevent cross-population or cultural behavior comparisons.
Trattner et al. [47], Li [48], Gulla et al. [49])	Easily accessible and standardized for research.	Limit the study of how user diversity influences recommendation performance.
Studies using custom or simulated datasets	Allow control over user and contextual variables.	Limited sample size and often rely on simulated or survey-based data.
(e.g., Bundasak et al. [42], Naik [43],	Enable domain-specific studies (e.g., food, health, or elderly care).	Reduced representativeness of real-world populations.
Aramayo et al. [44])	Adaptable to specific experimental designs.	Results are difficult to generalize to broader contexts.
Studies addressing user diversity and cross-population comparison	Highlight the importance of user diversity and fairness in recommendations.	Lack datasets with explicit demographic or cultural information.
(e.g., Ekstrand et al. [39], Beel et al. [40],	Identify biases and performance differences across user groups.	No standardized approach for population-level evaluation.
Raza and Ding [41])		Limited understanding of algorithm generalization across populations.

## Data Availability

The raw data supporting the conclusions of this article will be made available by the authors upon request.

## Supplementary Materials

The following supporting information can be downloaded at: https://www.mdpi.com/article/10.3390/foods14213770/s1, Table S1: Dataset containing all first-course dishes included in the study. Each entry includes the dish name, associated category tags, and detailed nutritional information. Table S2: Dataset containing all second-course dishes included in the study. Each entry includes the dish name, associated category tags, and detailed nutritional information. Table S3: Dataset containing all dessert dishes included in the study. Each entry includes the dish name, associated category tags, and detailed nutritional information.

## Abbreviations

The following abbreviations are used in this manuscript:

MAB	Multi-Armed Bandit
SARSA	State–Action–Reward–State–Action
DQN	Deep-Q Network
RL	Reinforcement Learning
FL	Fuzzy Logic
RS	Recommendation Systems
MPD	Markov Decision Process
DNN	Deep Neural Network
LSTM	Long Short-Term Memory
